# The Impacts of Land Use Patterns on Water Quality in a Trans-Boundary River Basin in Northeast China Based on Eco-Functional Regionalization

**DOI:** 10.3390/ijerph15091872

**Published:** 2018-08-29

**Authors:** Peixuan Cheng, Fansheng Meng, Yeyao Wang, Lingsong Zhang, Qi Yang, Mingcen Jiang

**Affiliations:** 1 Beijing Key Laboratory of Water Resources & Environmental Engineering, China University of Geosciences (Beijing), Beijing 100083, China; cheng_peixuan@126.com (P.C.); cen176624469@163.com (M.J.); 2 Chinese Research Academy of Environmental Sciences, Beijing 100012, China; mengfs@craes.org.cn (F.M.); zlingsong@163.com (L.Z.); 3 China National Environmental Monitoring Center, Beijing 100012, China; yeyaowang@163.com

**Keywords:** land use patterns, water quality variations, eco-functional regions, partial least square regression

## Abstract

The relationships between land use patterns and water quality in trans-boundary watersheds remain elusive due to the heterogeneous natural environment. We assess the impact of land use patterns on water quality at different eco-functional regions in the Songhua River basin during two hydrological seasons in 2016. The partial least square regression indicated that agricultural activities associated with most water quality pollutants in the region with a relative higher runoff depth and lower altitude. Intensive grazing had negative impacts on water quality in plain areas with low runoff depth. Forest was related negatively with degraded water quality in mountainous high flow region. Patch density and edge density had major impacts on water quality contaminants especially in mountainous high flow region; Contagion was related with non-point source pollutants in mountainous normal flow region; landscape shape index was an effective indicator for anions in some eco-regions in high flow season; Shannon’s diversity index contributed to degraded water quality in each eco-region, indicating the variation of landscape heterogeneity influenced water quality regardless of natural environment. The results provide a regional based approach of identifying the impact of land use patterns on water quality in order to improve water pollution control and land use management.

## 1. Introduction

Water quality integrates important geomorphic, hydrologic, and some of the biological processes of a watershed which make it one of the essential elements of a healthy watershed [1]. The deterioration of surface water quality is a considerable issue in river basin management throughout the world, which has become a serious threat to the chemical integrity of the aquatic environment. Surface water can be polluted by anthropogenic activities in two ways: (1) by point sources, such as sewage treatment discharge; and (2) by non-point sources such as overland runoff from urban and agricultural areas (buffer zones) [2]. Non-point source pollution is more difficult to verify than point sources due to the intricate and diffuse nature of the interactions between runoff and landscape [3]. Land use patterns have been deemed as a significant regulator of contaminants in surface flows and interflows, which make it a critical research topic for clarifying the correlations of surface water quality and non-point source pollutants [4]. Previous studies have reported about the relationship between water quality and the composition of land use types, such as cropland and urban, were related with stream pollutants positively, while forest and grasslands that were less influenced by anthropogenic activities had negative correlations [5,6]. The spatial configuration of landscapes in the watershed played an important part in identifying hydrological processes, natural habitats, energy flows, and nutrient cycles [7]. Thus, the variation of landscape is one of the main factors affecting non-point pollution. A large numbers of landscape metrics, for example the quantification of the landscape, have been developed to characterize landscape patterns and used for clarifying the linkages between the landscape and the water quality [7,8,9]. Previous research usually correlated surface water quality with either simple measures of land use types or landscape configuration in a watershed. However, either of them are not comprehensive enough to indicate the influences of land use management on water quality in a watershed. Therefore, conducting an analysis on the relationships between water quality indicators and both watershed land use types and landscape characteristics can produce a more comprehensive result. Generally, multivariate linear regression (MLR) has widely been used to quantify the relationships between land use patterns and water quality parameters, especially stepwise multiple regression [7,10,11]. However, classical regression approach presents several problems when analysing the relationships between land use/landscape metrics and water quality parameters. First, many land use types and landscape metrics are highly correlated, which produces redundancies and leads to inaccurate results. Additionally, sample size (the number of different study plots or individuals) should be larger than the number of predictors in order to assure the significance of the regression analysis [12]. Thus, the application of technique such as partial least-square regression (PLSR) can overcome the inherent limitations caused by classical multivariate regression when handling multi-collinear and noisy data [13]. PLSR is a method to analyse the response variable by using a set of independent variables having best predictive power [14]. The output of PLSR is a combination and generalization of the principal component analysis (PCA) technique and the multiple linear regression technique. PLSR can deal with variables that are highly collinear by explicitly assuming dependency among the variables and evaluating the underlying structures and is specifically suitable for cases in which the number of samples is less than the possible variables [15].

The Songhua River basin plays an important role in industry, agriculture and forestry. Intense anthropogenic activities such as agricultural production and urbanization have long been affecting the water quality of Songhua River. In order to obtain the goal of national water environment treatment, the regionalization of freshwater ecological functions has been carried out in the eight major watersheds in China including the Songhua River. The definition and method of water eco-functional regionalization was first proposed by Omernik [16]. In the past few decades, many countries have conducted their study on water eco-functional regionalization. Most states in the USA have already completed the IV level water eco-functional regionalization. In addition, EU has established the regionalization systems of different scales in the WFD, of which the macroscale eco-functional regionalization is based on the similar descriptions of typology, biology and ecology. China started to carry out the study on freshwater eco-functional regionalization during the Eleventh Five-year Plan with the support of National Major Science and Technology Program for Water Pollution Control and Treatment of China. It is of great importance to implement watershed management on the basis of eco-functional regionalization. Thus, the objectives of this study are as follows: (1) analyse the spatial and temporal distribution characteristics of the surface water quality in each eco-region; (2) quantify the relationships between land use/landscape characteristics and water quality parameters using PLSR in a sub-basin scale, and identify the main factors determining the water quality during normal flow and high-flow periods in each eco-region [17].

## 2. Materials and Methods

### 2.1. Study Area and Sampling Sites

The Songhua River Basin (41°42′~51°38′ N, 119°52′~129°31′ E) is located in the northeast of China and is one of China’s seven major river basins. This river basin occupies a large part of Heilongjiang Province, Jilin Province and the Northeastern part of Inner Mongolia Autonomous Region with an area of 55.7 × 104 km^2^. The Songhua River Basin is a trans-boundary watershed consists of three sub river basins: the Nenjiang River Basin in the North, the Second Songhua River Basin in the south, and the lower Songhua River Basin (the mainstream of Songhua River) in the northeast. Trans-boundary watershed is a watershed that crosses at least one political border, either border within a nation or an international boundary. The Nenjiang River originates on Yihehuli Mountain in the Great Khingan Mountains while the Second Songhua River originates at Tianchi Lake in the Changbai Mountains. Then, the two rivers travel all the way down and meet in Songyuan in Jilin Province. With a gentle slope and wide surface, the lower Songhua River carries the combined flow from Nenjiang and Second Songhua rivers, flowing northwestward 939 km before entering the Amur River.

The Songhua River Basin lies within a north temperate monsoon climate zone, and the temperature and rainfall varies significantly during the year, with the warmest month being July (20 °C~25 °C) and the coldest January (−20 °C). Annual precipitation averages 500 mm, showing a spatial tendency of higher in mountain area and lower in plain. 60~80% of the annual precipitation occurs from July to September, and only 5% occurs from December to February [18]. The Songhua River Basin is an important agricultural and industrial area in Northeast China. The dominant crops in the basin include soybean, corn, sorghum, and wheat. Industries mainly include petrochemical, machine manufacturing and paper making, distributed in the urban belt of main industrial cities like Harbin, Changchun, and Jilin along Songhua River [19].

**Figure 1:**
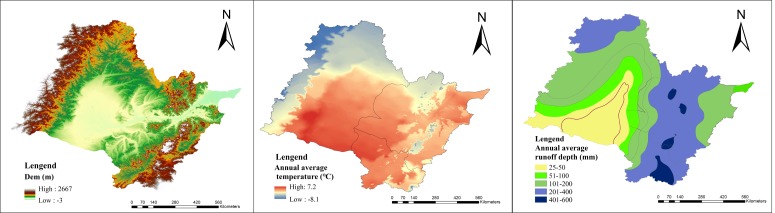
Distribution of environmental characteristics of Songhua River Basin.

In order to eliminate the impact of land use patterns on water quality covered up by heterogeneous natural environment elements, all 86 sampling sites were classified according to the level I water eco-functional regionalization of Songhua river basin. This regionalization grouped the 86 study sites into five different eco-regions: (1) mountainous normal flow (*n* = 10); (2) plain low-flow (*n* = 23); (3) hilly high flow (*n* = 32); (4) mountainous high flow (*n* = 12); (5) plain normal flow (*n* = 9). The five eco-regions were identified using a combination of climatic, hydrologic and topographic factors. The three factors referred to annual temperature, elevation and multi-average runoff depth respectively. Figure 1 shows the characteristics of the three factors. They were normalized and processed in ArcGis 10.2 (Esri, Redlands, CA, USA) by using the cell statistics and raster calculator in the spatial analysis toolset [20]. The main environmental characteristics of these five eco-regions were shown in Table 1.

### 2.2. Water Sampling and Analytical Methods

**Figure 2:**
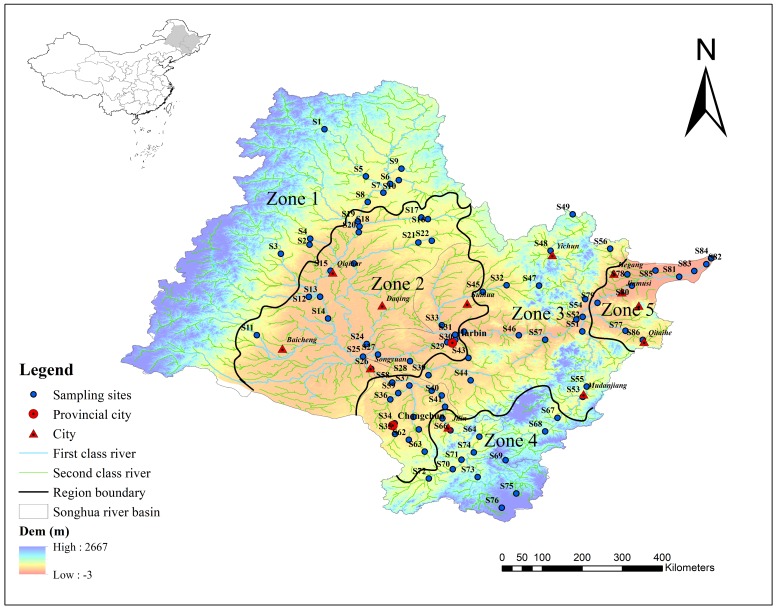
Locations of sampling sites in Songhua River Basin.

Water sampling was conducted monthly at 86 sites throughout the Songhua river basin in high-flow season (July), normal flow season (September) in 2016 and in icebound season (March) in 2017 (Figure 2).

Forty-three of those sample sites are part of the local government’s long term monitoring network (Table A1). Not all samples were collected during the icebound season as some of the streams were frozen to the bottom. The water samples at each sites were sampled in polyethylene bottles pre-rinsed three times with distilled water and kept below 4 °C for laboratory analysis. Three samples were collected at each sampling site and a total of 256 samples were collected. Twelve representative parameters were selected for measurement, which are important indicators of water contamination influenced by anthropogenic activities. The parameters included pH, electrical conductivity (EC, µs·cm^−1^), dissolved oxygen (DO, mg·L^−1^), chemical oxygen demand (COD, mg·L^−1^), permanganate index (COD_Mn_, mg·L^−1^), ammonia nitrogen (NH_3_-N, mg·L^−1^), nitrate nitrogen (NO_3_-N, mg·L^−1^), total nitrogen (TN, mg·L^−1^), total phosphorus (TP, mg·L^−1^), fluoride (F^−^, mg·L^−1^), chloride (Cl^−^, mg·L^−1^), and sulfate (${\mathrm{SO}}_{4}^{2-}$, mg·L^−1^). The values of pH, DO and EC were directly measured in situ with a multi-parameter water quality monitoring instrument (Thermo Fisher Scientific, Waltham, MA, USA). The values of other parameters mentioned in the paper were analyzed in the laboratory following the national standard methods [21]. Additionally, water samples except for fluoride, chloride and sulfate analysis were acidified with sulfuric acid to adjust the pH < 2.

### 2.3. Land Use and Landscape Metrics

**Figure 3:**
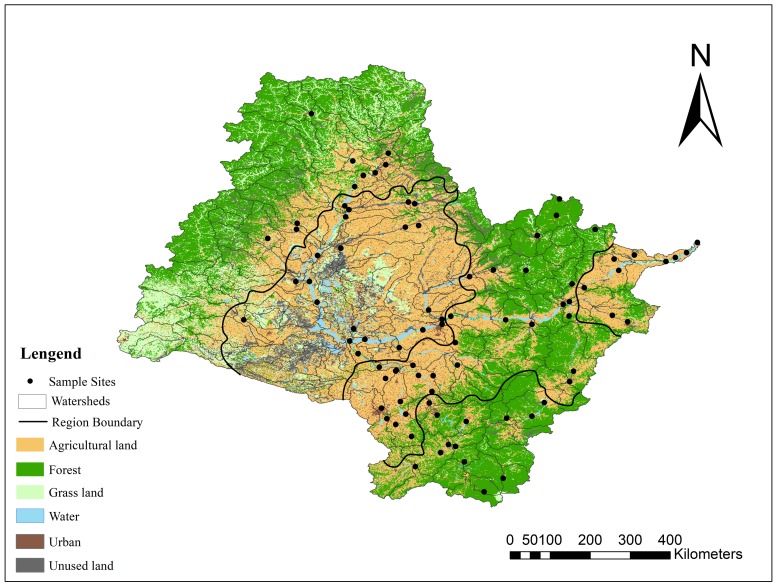
Land use distribution in Songhua River Basin.

The stream network and sub-basin boundaries were extracted from a digital elevation model (DEM, 30 m × 30 m data) using the hydrology toolset in ArcGIS 10.2. As a result, there were a total of 144 sub-basins formed in the study area, 53 sub-catchments were selected to study the relationships between land use patterns and water quality. Land use patterns were acknowledged to have little change within 5 years. Thus we chose the land use data of Songhua river basin in 2015, which was provided by Data Centre for Resource and Environmental Sciences, Chinese Academy of Sciences (RESDC) (http://www.resdc.cn). The land use types were classified into six categories: (1) agricultural land, mostly planted with corn, rice and soybean; (2) forest; (3) vegetated land; (4) water bodies, including rivers, reservoirs and ponds; (5) urban areas, including residential, commercial and industrial lands; and (6) unused land, including gravel, bare ground, and bare rock (Figure 3). The six land use types were commonly used in previous studies and should all be considered to better understand the land use pattern and its relationship with water quality in the Songhua River Basin [5,7,22]. In order to investigate the relationships between landscape configurations and water quality, the landscape metrics in 53 sub-catchments representing the patch size, shape, structure, and landscape diversity were chosen at the landscape levels (Table 2). They have been commonly used in previous studies in dealing with land use patterns in explaining water quality. In addition, these landscape metrics are important in understanding the ecological functioning and human perception in a landscape [8,23,24]. FRAGSTATS 4.0 (University of Massachusetts: Amherst, MA, USA) was used to calculate the landscape metrics based on land use data.

### 2.4. Statistical Analysis

All the water quality data were tested for normality by eco-regions using the Shapiro-Wilk test, since the number of sampling sites in each eco-region was below 50. Parameters not normally distributed were log transformed to increase variable normality [25]. One-way analysis of variance (ANOVA) with the post hoc Tukey’s test was used to compare water quality variations between different eco-regions and seasons at significance level of *p* < 0.05. Boxplots of all sampling sites in the five eco-regions for the 12 water quality parameters were performed to study their seasonal and spatial variability. ArcGIS 10.2 was used to map the distributions of concentration of each water quality parameter by spatial interpolation in order to better understand the variation of water quality among sites. Besides water quality, one way ANOVA with the post hoc Turkey’s test was used to compare the variance of landscape metrics between different eco-regions. Welch’s Anova test was also done in case the heterogeneity of variance appears. Both ANOVA test were done at the significance level of *p* < 0.05. The test of normality of the landscape metrics was done using the Shapiro-Wilk test initially.

The partial least square regression (PLSR) was used to explore the relationships between land use patterns and water quality variations in different eco-regions and identify the key predictors for degraded water quality. This was carried out by converting explanatory variables onto orthogonal ‘latent’ components, which stand for independent variables in a regression. The calculated latent components in PLSR maximize the covariance between the response and explanatory variables through the simultaneous decomposition of X and Y matrices of vectors. The PLSR models were performed in SIMCA-P [26]. Cross-validation was the criterion used to determine the minimum number of latent components needed to acquire the most predictive PLSR model.

Within SIMCA-P, Q^2^ (the fraction of the total variation of the dependent variables that can be predicted by a component) stands for the cross-validation of components, when Q^2^ is larger than 0.5, the model is expected to introduce good predictive ability, while indicating no significance when Q^2^ is smaller than 0.05. Q^2^ was computed using the equation below:

(1) $$Q^{2}=1.0-PRESS\;/SS$$

where *PRESS* is the abbreviation for the predicted residual sum of squares, and *SS* stands for residual sum of squares.

In the PLSR modelling, the variable importance in projection (VIP) is a criterion of estimating which independent variables can elucidate the dependent variables most significantly. VIP is calculated by the following equation:

(2) $$VIP_{j}={\left\{p\sum_{h=1\;}^{m}\sum_{k}R^{2}\left(y_{k},t_{h}\right)w_{hj}^{2}/\sum_{h=1}^{m}\sum_{k}R^{2}\left(y_{k},t_{h}\right)\right\}}^{1/2}$$

In Equation (2), *p* is the number of independent variables, *m* represents the number of components extracted from independent variables, *k* stands for the number of dependent variables, *t_h_* represents the components of independent variables, *R*^2^(*y_k_*,*t_h_*) represents the square of regression coefficients of *y_k_* and *t_h_*, $w_{hj}^{2}$ is the weight of independent variables contributing to component *t_h_*.

It is generally recognized that the independent variables with VIP values above 1 are of great significance for dependent variables; variable with VIP values below 0.8 are of minor importance; it is of medium significance when VIP values are between 0.8 and 1. The regression coefficients indicate the direction and strength of the impact of each variable in the PLSR model [14]. In order to avoid over fitting which leads to low statistical significance PLSR models for each response factors, not all anthropogenic factors must be included in a PLSR model. Therefore, the following PLSR analysis procedure was followed to obtain a most predictive model. Firstly, for each given water quality parameter, all predictor variables were included in the model. Next, a series of new PLSR models were conducted in which each new PLSR process was implemented with a variable excluded in order to minimize the value difference between the explained variation in the response (R^2^) and the predictive ability of the model (Q^2^) This procedure was repeated until as few predictors were remained [27]. Finally, the PLSR model with the largest Q^2^ was chosen as the optimal model. A test of collinearity of the explanatory variables was done in prior of the application of PLSR method (Table A2).

## 3. Results

### 3.1. Characteristics of Water Quality

**Figure 4:**
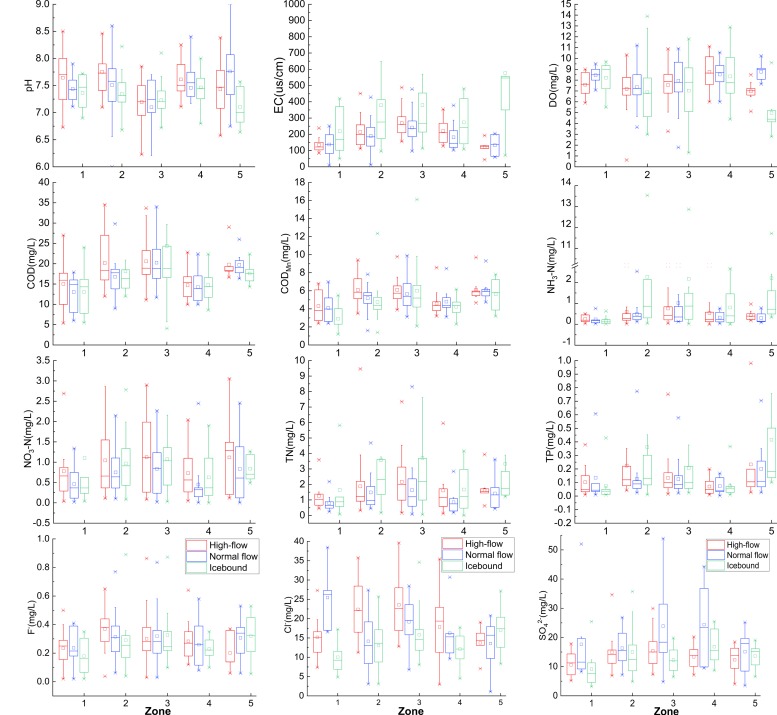
Distribution patterns of water quality parameters during high-flow, normal flow and icebound seasons in different eco-regions of the Songhua River Basin, China (The box represented 25th and 75th percentiles; the small square represented mean; the line in box represented median; values above or below whiskers were outliers). Zone 1–5 refers to the five eco-region listed in Table 1.

**Figure A1:**
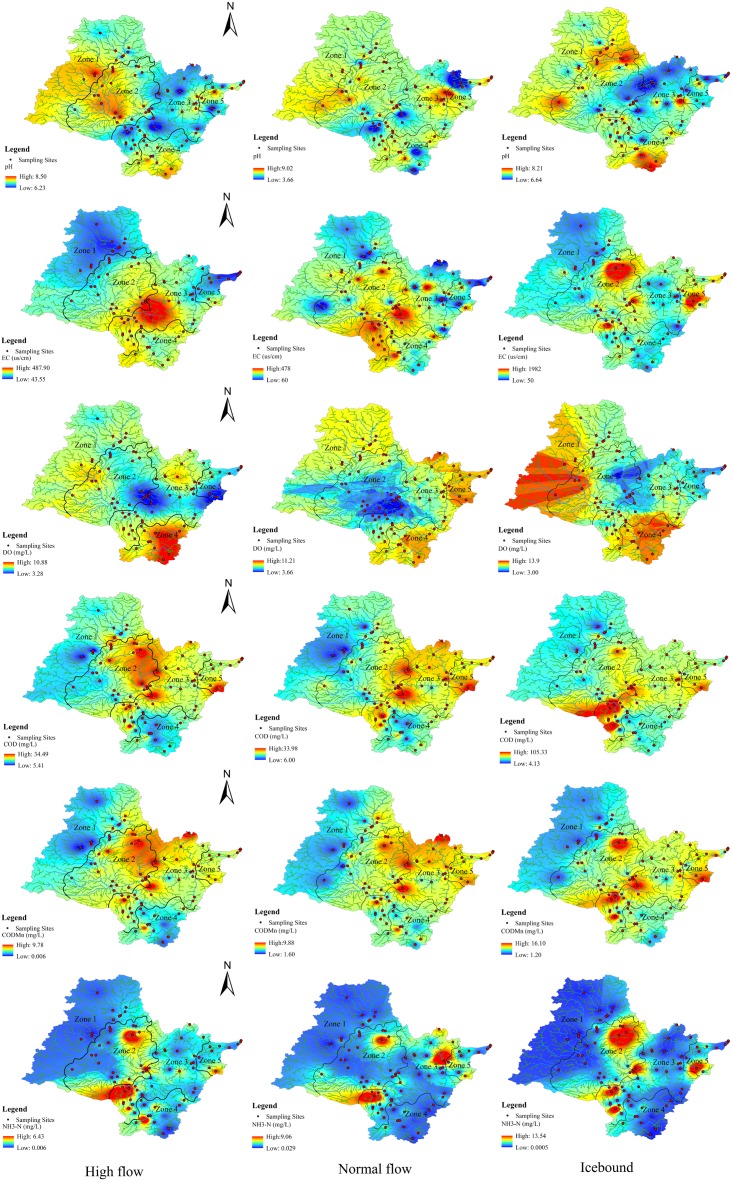

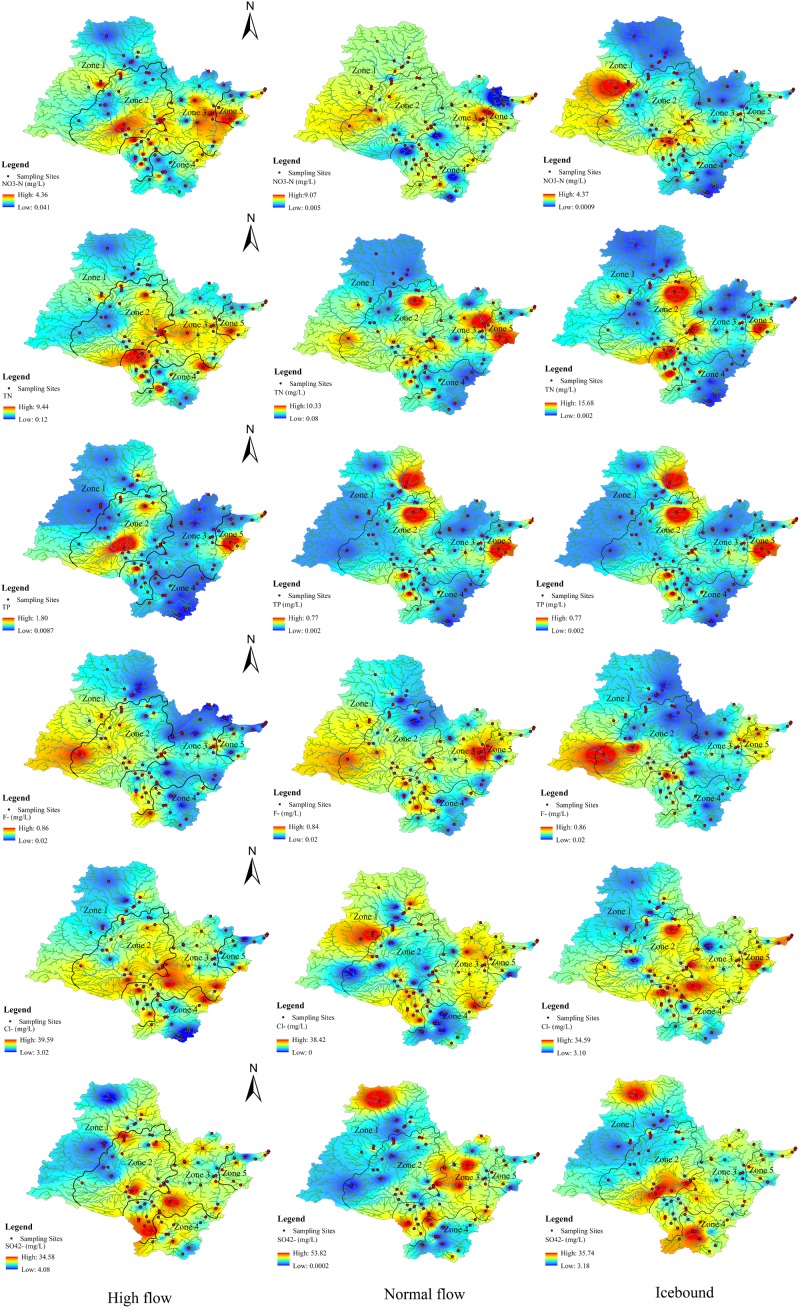
Spatial variations of water quality parameters during different seasons in Songhua River Basin.

Spatial and seasonal variations of water quality parameters in five eco-regions were illustrated by box-plots (Figure 4). Interpolation maps were illustrated by ArcGis 10.2 in order to help better understanding the spatial variations of water contaminants (Figure A1). According to the one-way ANOVA, all variables showed significant spatial differences among the five eco-regions (*p* < 0.05). Most of the variables showed significant temporal differences between different seasons. Large pH values often occurred in high-flow period, except in Zone 1 where high pH was found in normal flow period. EC was found higher in icebound season in every eco-region, however there was no significant difference between high-flow and normal flow period. The low concentrations of DO were mostly observed in ice bound season in every eco region, except in Zone 4. The concentrations of COD showed obvious temporal differences only in Zone 2 and Zone 5, large values were found in high-flow period in Zone 2, while higher concentrations were observed in Zone 5 in normal flow periods. The concentrations of COD_Mn_ were mostly found higher in high-flow periods, whereas low concentrations were found in the ice bound season, and only Zone 5 presents large values in the mean flow period. The concentrations of NH_3_-N were higher in ice bound season in every eco-region except in Zone 1. NO_3_-N had large values in high-flow periods while it presented low values in normal flow periods. The concentrations of TN were higher in the ice-bound season in every eco-region except for Zone 1. The values of F^−^ didn’t show obvious temporal variations, while the concentrations of Cl^−^ and ${\mathrm{SO}}_{4}^{2-}$ varied in different periods among the five eco-regions.

### 3.2. Land Use and Landscape Characteristics at Sub-Basin Scale

**Figure 5:**
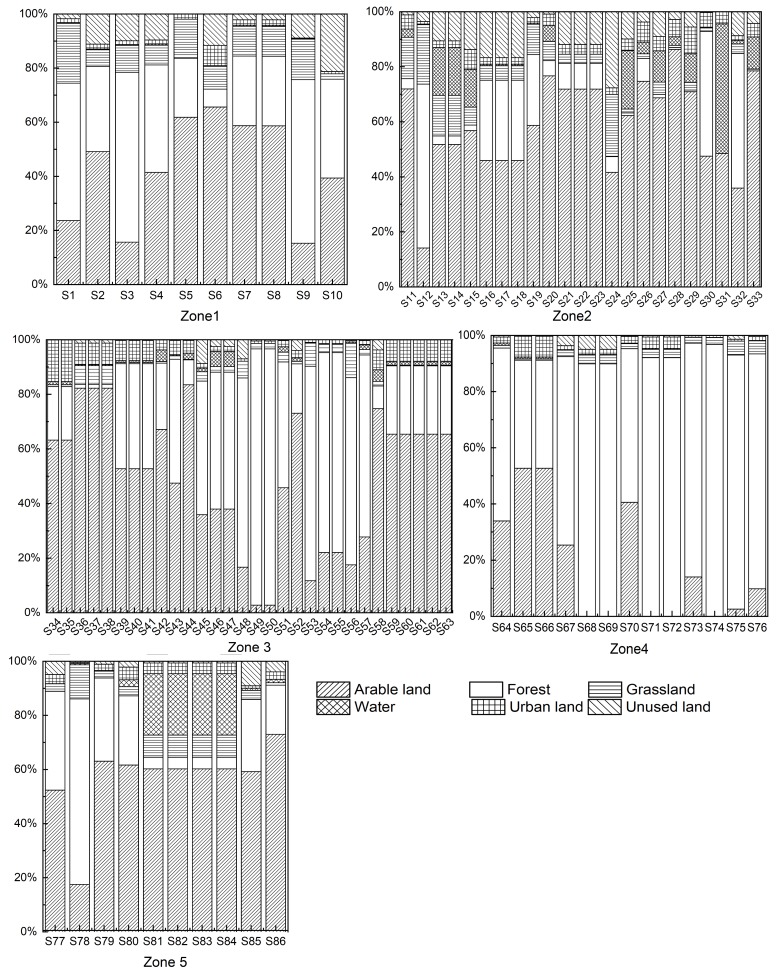
Land use composition (%) in five eco-regions in Songhua River Basin, China.

Figure 5 showed the distribution of land use for each sampling sites at sub-basin scale in 2015. Arable land and forest were the dominant land use in Zone 1, ranging from 15.26% to 65.60% and 6.61% to 62.78% respectively. A relatively less proportion of urban land use was occurred in Zone 1, except for S7 which occupied 7.52% of the sub-basin. Arable land was the dominant land use in Zone 2, ranging from 14.13% to 86.28%. Zone 2 had a relatively larger proportion of urban land use with an average of 4.06% and a maximum of 9.68%. However, the ratio of forest area was relatively lower than other eco-regions with a maximum of 59.52%, while others were lower than 10%. Therefore Zone 2 was obviously disturbed by anthropogenic activities. Arable land was the dominant land use in Zone 3, ranging from 2.76% to 83.46%, with an average of 50.62%. Likewise, a significant amount of forest was discovered in some of the sub-basins, ranging from 1.41% to 93.86% with an average of 38.96%. The proportion of urban land use was the highest among the five zones, with an average of 5.16% and a maximum of 15.26%. A large proportion of forest was observed in Zone 4, with an average of 64.94% and a maximum of 90.46%. The land use ratio of arable land ranked only second to forest, with a maximum of 29.05%. The proportion of urban land use was lower than other eco-region with an average of 2.44%. The dominant land use in Zone 5 was arable land, ranging from 17.53% to 63.02%, with an average of 54.95%. The ratio of forest in most of the sub-basins was lower than 30%, with an average of 30%, the highest was 68.55% in S79. The average proportion of urban land use was 3.08%.

The dominant land use types in Songhua river basin were arable land and forest. It was observed that sub-basins with a larger ratio of urban land had a lesser ratio of grassland and forest but a larger ratio of arable land. This observation might indicate that urbanization on one hand had decreased the amount of forest and grassland to support more living residents in the city, on the other hand retained or even increased the amount of arable land for more food was needed.

According to the one-way ANOVA, most of the landscape metrics except for LSI and AI were showed significant variations among the five eco-region (*p* < 0.05) (Table 3). The highest value of PD (0.17/100 ha), LPI (78.77%), CONTAG (56.59%) and AI (81.29%) and the lowest value of PD (0.04/100 ha), SHDI (0.84), SHEI (0.32) and ED (3.63 m/ha) were recorded in Zone 3. The highest value of ED (10.76 m/ha), LSI (27.22), IJI (73.84%) and SHDI (2.04) and the lowest value of LPI (10.61%), LSI (2.76) and CONTAG (34.85%) were recorded in Zone 2. The highest value of SHEI (0.85) and the lowest values of IJI (41.82%) were recorded in Zone1. The lowest value of AI (0.52%) was observed in Zone 4.

### 3.3. Linkages between Water Quality Parameters and Land Use, Landscape Metrics in Each Eco-Regions

The PLSR approach was applied to quantify the relationship between water quality and land use/landscape metrics for each eco-region mainly in the high flow and normal flow seasons. The ice-bound season was not included due to the little surface runoff. The summary of each optimal PLSR models were provided in Table 4, including the R^2^ and Q^2^ of each model as well as the number of components extracted in each model was to reach the minimum difference between R^2^ and Q^2^ and a larger Q^2^. The value of Q^2^ should be larger than 0.5 to make the model predictive and significant. As Table 4 shows, most of the optimal models for water quality parameters extracted two components in both seasons, whereas certain models for water quality parameters extracted three components. In addition, individual models for water quality parameters only extracted one component in some eco-regions which leaded to low values of Q^2^. As we had observed, in Zone 1, Zone 2 and Zone 3, the optimal model for ${\mathrm{SO}}_{4}^{2-}$ in high flow seasons and the models for Cl^−^ in Zone 4 in both seasons, as well as the model for Cl^−^ in high flow season extracted only one component. This indicate that increasing the number of components to the PLSR models cannot continually improve the explained variance and leads to lower predictive ability (i.e., larger gap between model R^2^ and Q^2^ values), the subsequent components are not strongly correlated with the residuals of the predicted variable [12]. Therefore, the models for ${\mathrm{SO}}_{4}^{2-}$ in Zone 1, Zone 2 and Zone 3 in high-flow season and the models for Cl^−^ in Zone 4 in both seasons and in Zone 5 in high-flow season were of low significance and predictive power. 

**Figure 6:**
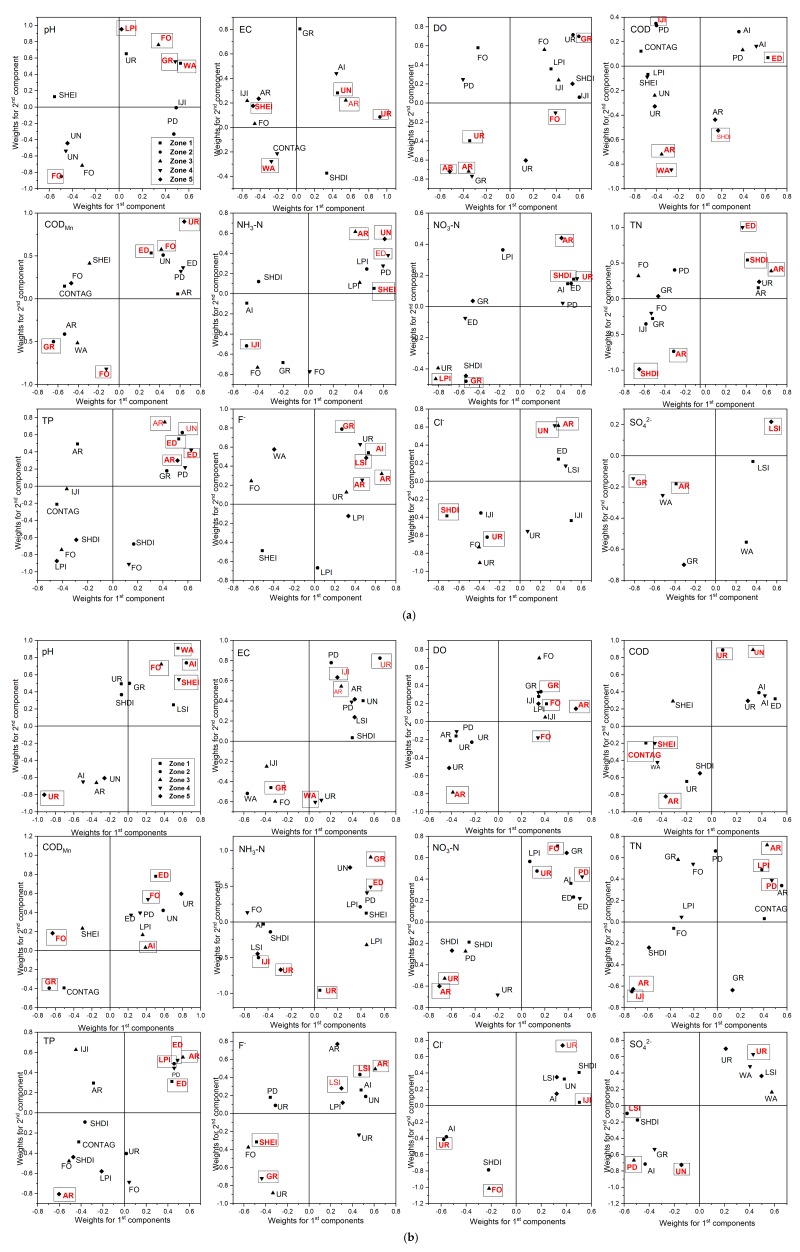
(**a**) Weight plots of the first and second PLSR components for individual water quality parameter in the high-flow season, and (**b**) weight plots of the first and second PLSR components for individual water quality parameter in the normal flow season. Land use/landscape variables with the highest VIP values in each eco-region was in red and highlighted with boxes. Abbreviations for land use/landscape metrics are listed in Table 2.

The regression coefficient (RC) and the variable importance for the projection (VIP) are intuitive and comprehensive expressions of the relative importance of the variables when indicating how important land use types and landscape metrics are to the specific water quality parameters. Table 5 and Table 6 illustrated the key variables (VIP > 1) of each optimal model with their regression coefficients (RCs) in high-flow and normal flow seasons respectively. Table A3 and Table A4 presented all the regression coefficients of all the explanatory variables in each optimal model. The PLSR weights can be used to better understand the quantitative relation between the explanatory variables and response, because they are linear combinations of the original variables that define the scores. It is another important symbol to indicate the importance of individual land use/landscape metrics to water quality parameters. The weight plots illustrated the key predictors (VIP > 1) of each optimal model and highlighted the predictors with the highest weights in each model (Figure 6).

In Zone 1, water quality variables were influenced mostly by landscape metrics during high-flow and normal flow seasons. During high-flow season, only pH, EC, DO and ${\mathrm{SO}}_{4}^{2-}$ were most influenced by land use metrics, of which the key variables with highest VIP values were WA UN, UR and AR respectively. The key variables with the highest VIP values for other water quality contaminants were mostly landscape metrics. ED was the most important predictor for COD, COD_Mn_ and TP, while SHDI contributed the most to NO_3_-N, TN and Cl^−^. NH_3_-N appeared to be influenced the most by SHEI, whereas AI was the most predictive variables of F^−^. During the normal flow season, the most important predictors for pH and EC were the same as the high-flow season, while DO was most influenced by FO instead of UR. The key variables with the highest VIP values for NH_3_-N and NO_3_-N were UR and FO respectively. ED was the most important predictor for COD_Mn_ and TP, while CONTAG contributed the most to COD. LPI, SHEI and IJI were the most important predictors for TN, F^−^ and Cl^−^ respectively.

In Zone 2, land use metrics predominated in the key variables with the highest VIP values in optimal models. During high-flow season, GR was the most important variables of the variations of DO, COD_Mn_, NO_3_-N, F^−^ and Cl^−^, while IJI contributed the most to COD and NH_3_-N. FO, UR, AR and UN were the most important predictors for pH, EC, TN and TP. During normal flow season, pH, DO and COD_Mn_ were all most influenced by GR, whereas UR contributed most to EC, COD, NO_3_-N and Cl^−^. Landscape metrics such as IJI, LSI and LPI were observed as the most important indicators of the models (VIP > 1). Lower IJI contributed to higher NH_3_-N and TN; higher LPI dedicated to higher TP, while LSI was positively correlated with F^−^, but negatively correlated with ${\mathrm{SO}}_{4}^{2-}$.

In Zone 3, land use metrics contributed to more water quality variables than landscape metrics. During high-flow season, all water quality parameters were most influenced by AR, except for pH, COD_Mn_ and NO_3_-N. FO was the most important predictors for pH and COD_Mn_, while LPI contributed the most to NO_3_-N. During normal flow season, AI and PD were observed as the most important predictors for COD_Mn_ and ${\mathrm{SO}}_{4}^{2-}$, whereas other water quality parameters were most influenced by land use metrics.

In Zone 4, during high-flow season, pH and ${\mathrm{SO}}_{4}^{2-}$ were most influenced by GR, whereas WA contributed the most to EC and COD. FO was observed the most important predictor for DO and COD_Mn_, and was positively related to DO and COD_Mn_. UR, AR and UN contributed most to NO_3_-N, F^−^ and Cl^−^ respectively. The rest of the water quality parameters were most influenced by ED, which was the only landscape metrics with the highest VIP values. During the normal flow season, landscape metrics such as SHEI, ED and PD contributed more to water quality parameters than that in high-flow season. SHEI was the most important variable for pH and COD; ED contributed more to NH_3_-N and TP; NO_3_-N and TN was impacted most by PD. Other water quality parameters were contributed most by land use metrics. The most important predictor for DO and COD_Mn_ was FO which was the same as high-flow season. UR contributed the most to EC and ${\mathrm{SO}}_{4}^{2-}$, while F^−^ was most influenced by GR.

In Zone 5, during the high-flow season, AR was the most significant predictor for DO, NO_3_-N and TP; COD_Mn_ and NH_3_-N were most impacted by UR and UN respectively. The rest of the water quality parameters were influenced by landscape metrics in most cases. SHDI contributed most to COD and TN, while F^−^ and ${\mathrm{SO}}_{4}^{2-}$ were most impacted by LSI; LPI and SHEI was the most significant predictors for pH and EC respectively. During the normal flow season, F^−^ was most influenced by LSI, whereas pH and Cl^−^ were contributed most by UR. FO and UN were the most significant predictors for COD_MN_ and ${\mathrm{SO}}_{4}^{2-}$ respectively. Other water quality parameters were most influenced by AR.

It should be noted that all the predictors in the final optimal model were to some extent correlated with the specific water quality parameter. However only predictors with VIP values above 1 were considered to be of major importance. Overall, it was observed that the key variables with highest VIP values of the optimal models were spatially and temporally different. In addition, landscape metrics contributed more in Zone 2, Zone 3 and Zone 4, while land use metrics dominated in the other two eco-regions.

## 4. Discussion

### 4.1. Key Land Use Types Predicting Water Quality

Many studies have reported that agricultural and urban land uses contribute to degrading water quality in adjacent aquatic systems, whereas vegetated areas such as grassland and forest have a positive contribution to water quality [6,28]. The results of this study were generally consistent with such previous findings. However not all water quality parameters in each eco-region showed a strong relationship with agricultural land use and urban land use.

In the high-flow season, although arable land was not always the variable with the highest VIP values in the optimal models of water quality indicators, it was predictive and associated with most water quality variables in each eco-region mainly in Zone 3 and Zone 5. Arable land was positively correlated with organic matter and some of the nutrients, while it correlated negatively with DO. This indicated that arable land actually served as a source for pollution in these eco-regions. Previous studies had noted that arable land had a positive impact on degraded water quality due to agricultural activities, such as fertilizer and pesticide application as well as livestock raising, which were often the major non-point source [4]. Forest was found to be closely related to most water quality variables than other land use types in Zone 4. It was identified that forest was positively correlated with DO and COD_Mn_ during both seasons, while negatively correlated with NH_3_-N, TN and TP. It was unexpected that higher forest land use contributed to higher COD_Mn_, which to some extent contradicted previous research. Therefore, a possible speculation of this phenomenon is that the increase of forest would cause the accumulation of refractory organic matter derived from decaying plant material, afterwards flows into the surface water with overland runoff [29,30,31]. In addition, the terrain in Zone 4 is steeper than that in other eco-region which means that higher slope exists in this region. Previous study claimed that with an increasing slope, higher water flow rates would contribute to soil erosion and to the rates of particulate matter that picks up pollutants [32]. Although the dominant land use type in Zone 2 was arable land, the land use type contributed the most to the water quality in this region came out to be grassland. A negative relationship was identified between grassland and COD_Mn_, NO_3_-N, TP and F^−^, while DO was found positively correlated with grassland, which indicated the fixation and absorption effects of grassland for pollutants in overland runoff [5]. However the concentrations of non-point source pollutants, such as COD_Mn_ and TN in Zone 2 were relatively high among the 5 eco-regions and was appeared to be related positively with arable land. This suggested that effect of grassland in decontaminating overland runoff has been weaken, even in upstream of the sub-basin. The main leading might be the looseness of surface soil structure caused by intensive grazing, which had been discovered frequently throughout our field survey. In addition, Zone 2 was known as the black soil area, the rich humus in the soil contributed to the increase of COD_Mn_ and TN in surface water, especially in rainy season.

In the normal flow season, arable land was no longer the variable associating with the most water quality parameters in Songhua River Basin. Forest and urban land use had become the main predictors for water quality in some eco-regions such as Zone 1 and Zone 4. This phenomenon should be attributed to the decrease of precipitation, which reduced the volume of overland runoff generated by arable land. Another reason might be the first flush effects in storm events, which made surface runoff pollutants entering rivers more apparently in high flow season than that in other seasons [33]. Thus, fewer non-point pollutants was transported into surface water in normal flow season. However, forest was known to have the ability of intercepting the degraded water and filtering out nutrients. Thus forest land uses became one of the most influential factors towards the concentration of nutrients. Urban land use revealed its contribution to COD and NH_3_-N indicated that the water quality in Zone 1 was polluted by possible point sources mainly from domestic and industrial sewage since these pollution sources mostly distributed in built-up areas [5,7,32]. TN was the only water quality parameter that was dominated by arable land in Zone 2, while others were contributed mostly by grassland and urban land use. Indicating that point sources were more likely to be the major source of water contamination compared to that in high-flow season as urban areas was mostly covered with impervious surface and the drainage was continually routed to wastewater treatment plants and then discharged to local rivers as point sources [34].

### 4.2. Key Landscape Metrics Predicting Water Quality

Anthropogenic activities not only influence the composition of land use, but also change the landscape pattern. Previous studied had indicated that non-point source pollution loading, including soil erosion, sediment, and nutrient runoff, in rural watersheds was closely relevant to the landscape structure [35]. For example, a previous study had reported that landscape metrics consistently explained a 65% to 86% of the total variation in nutrients and suspended sediment to stream [36]. Landscape pattern metrics have been developed through the use of spatial tools, in order to attain the goal of quantification land use patterns and understand spatial heterogeneity and landscape structure [37].

In this study, degraded water quality was positively related with landscape fragmentation metrics such as PD and ED and was negatively related with LPI in most eco-regions. Previous studies have reported that PD and ED showed positive relationships with TN, TP, COD, and BOD concentrations in reservoirs in South Korea which was dominated by vegetated areas [7]. That was consistent with what it was found in this study, especially in Zone 1 and Zone 4, which are both mountainous regions with large proportions of forest. The value of PD and ED increased when growing numbers of small patched land cover types appeared in the watershed. The degree of forest fragmentation could be reflected by PD and ED [13]. Therefore, highly fragmented forest might not function efficiently to increase the permeation and decrease overland runoff and erosion from agricultural and urbanized areas, consequently let pollutants, sediments and nutrients flowed into the surface water without efficient interception.

The CONTAG metric reflects the level of aggregation of land use types. Generally, a low CONTAG metric value means that land use types are highly fragmented, while a high value of CONTAG represents an aggregated landscape [38]. In this study, CONTAG was observed to be negatively related with non-point pollutants in the mountainous normal flow region (Zone 1) in both high-flow and normal flow seasons. Previous research had reported that in streams non-point pollutants are mainly derived from soil erosion and sediment yield [13]. Thus the CONTAG metric might be a factor contributing to soil erosion and sediment yield, especially in mountainous areas. In addition, intensive soil erosion occurred in the eastern part of the mountainous normal flow region. Thus, land use management in this region should focus on the aggregation of land use types in order to reduce in-stream non-point pollutants.

The AI metric represents the degree of physical connectedness and aggregation of land use within watersheds, and it is higher when land uses are more clustered and aggregated. In this study, the AI metric contributed to more water quality parameters in the normal flow season, and a negative relationship was identified between AI and water quality in two seasons. Therefore, degraded water quality usually occurs in watersheds with scattered land uses and plentiful land use patches.

The IJI metric represents the degree to which patch types are interspersed (not necessarily dispersed); lower values represent landscapes with poorly interspersed patch types (i.e., uneven distribution of patch type adjacencies), whereas higher values characterize landscapes in which the patch types are well distributed (i.e., equally adjacent to each other). The IJI metric was a more effective indicator for water quality parameters in high-flow season, which was positively correlated with COD, nutrients, F^−^ and Cl^−^, while negatively correlated with DO. This suggested that human activities might disperse patches and therefore increase the potential of contaminants flowing into the river.

The LSI metric is an alternative to patch shape indices based on the average patch characteristics, which measures the perimeter-to-area ratio for landscape as a whole [38]. The higher the index indicates the more complex of the patches in the landscape. In this study, the LSI metric was an effective indicator for water quality variables of F^−^, Cl^−^ and ${\mathrm{SO}}_{4}^{2-}$ in high-flow season in Zone 1, Zone 4 and Zone 5, and pH, EC and NH_3_-N were found positively related to LSI in normal flow season in Zone 1 and Zone 5, besides F^−^ and ${\mathrm{SO}}_{4}^{2-}$. This suggested that the shape of the patches in the landscape contributed more to anions concentration. Higher LSI indicates more edges presented in a landscape, which leads to negative impacts on water quality.

The SHDI and SHEI are diversity metrics influenced by richness and evenness respectively. Richness refers to the present patch type’s number; evenness refers to the distribution of area among different types [38]. Larger values of SHDI and SHEI imply greater landscape diversity. Although SHDI and SHEI were not always the predictors with the highest VIP, they were both positively correlated with degraded water quality in the two seasons. The SHDI metric indicated more water quality variables in each eco-region than SHEI, except in Zone 3 and Zone 4 which only SHEI was observed contributing to water quality variables. This suggested that the degradation of water quality is likely to occur when the number of land use types increased and when different land use types are distributed homogenously.

### 4.3. Importance of an Eco-Functional Regionalization Land Use Pattern Impacts

The results of this study demonstrated that the key predictors and seasonal variations of the optimal models of each eco-region were considerably different (Table 4, Table 5 and Table 6). It was reported in previous researches that the relationship between land use pattern and water quality to some extent was influenced by regional differences because the gradients of anthropogenic land use were frequently overlapped on an underlying gradient in primitive characteristics of natural terrain [39]. For instance, urban areas often located in flat land instead of mountainous region because the former is more likely to develop rapidly. This is consistent with the results in our study that a relatively high proportion of urban areas generally appears in the plain catchments. Allan emphasized that anthropogenic land uses often covary with natural landscape, a factor that can result in overestimation of land use influence on river water quality. In this study, the five eco-regions were classified based on an analysis involving climatic, hydrologic and topographic factors. The relationships between land use patterns and water quality were analyzed without the disturbance of natural terrain since each eco-region has the similar topographic features. The results showed that the significant predictors of water quality parameters in each eco-region were apparently different. In addition, previous study had proposed that landscape heterogeneity within a large river basin might bring in model errors which implied the influences of agricultural lands in hilly watersheds might be covered up by strong influence of urban areas in the plain watersheds [11]. This indicated the importance of regional basis when exploring the relationship between land use patterns and stream water quality. Statistical models should be conducted on a relatively homogenous region in accordance to natural terrain, climatic and hydrological condition in order to get a reliable result. In addition, it also meets the requirement of the Chinese government that the watershed environment should be administrated based on the eco-functional regionalization.

### 4.4. Model Performance and Limitations

Multicollinearity among various land use/landscape metrics is one of the obstacles when establishing precise relationship between land use patterns and stream water quality. PLSR is of great advantage in this study because it is particularly conducive when the number of predictive indicators is similar to or higher than the number of observations and/or there is strong collinearity among the predictors [12]. Additionally, the PLSR methodology to some extent excludes the confounding relationships among both the independent and dependent variables and encourages a more impartial view of the contribution of land use patterns to stream water pollutants [27]. Therefore, this approach could be applied to the investigation of water contamination in other watersheds.

The method used in this study has some limitations. First, the water quality data during the low-flow and snowmelt period was not collected. Some researchers have claimed that a low-flow period should also be involved in the analysis, as farming activities such as sowing and fertilization occur frequently during this period, which causes negative impacts on river water quality [4,5]. As for the snowmelt period, some researchers take it into account when studying the watersheds located in cold-climate regions, as nutrient export produced by snowmelt runoff may differ significantly from runoff during storm events [25,40]. Thus, it is desirable to involve in situ sampling in low-flow and snowmelt period when investigating the relationships between land use patterns and water quality in the Songhua River basin in order to better understand the temporal variability in the watershed located in cold climate region and with intense agricultural activities. Second, some of the models were of low predictive power within the five eco-regions. such as the models (Q^2^ < 0.5) of ${\mathrm{SO}}_{4}^{2-}$ in Zone 1, Zone 2 and Zone 3 in high-flow season and the models of Cl^−^ in Zone 4 in both seasons and in Zone 5 in high-flow season. This indicated that the variations of ${\mathrm{SO}}_{4}^{2-}$ and Cl^−^ might not be exclusively explained by land use and landscape metrics. This was consistent with some previous studies claiming that road density and geology characteristics could also contribute to the variation of ions besides land use patterns [10,41]. Therefore, further researches are inevitable to include other non-land use variables to build empirical models in order to obtain a higher predictive accuracy and confidence level.

## 5. Conclusions

The results of this study demonstrated that water contamination of the Songhua River basin in China presented high temporal and spatial variations within the five eco-regions. A partial least square regression (PLSR) approach was applied in exploring the relationships between land use patterns and specific water pollutants during both high-flow and normal flow seasons on a watershed scale. The results suggested that in different eco-regions, water quality parameters were influenced by different land use/landscape metrics. Arable land was observed a predictive variable contributing to degraded water quality mainly in hilly high flow region (Zone 3) and plain normal flow region (Zone 5), indicating that agricultural activities were the main factor contributing to degraded water quality in regions with a relative higher runoff depth and lower altitude. Grassland influenced most water quality parameters in the plain low flow region (Zone 2), suggesting the effect of intensive grazing in plain areas. Forest was observed to be negatively related to degraded water quality in mountainous high flow regions (Zone 4), indicating the importance of forest in mountainous areas. Landscape metrics were predictive variables contributing to degraded water quality in each eco-region during both hydrological seasons. The water quality degradation in both seasons were positively related to landscape metrics of patch density (PD), edge density (ED), landscape shape index (IJI), interspersion juxtaposition index (LSI), Shannon’s diversity index (SHDI) and Shannon’s evenness index (SHEI), and negatively associated with largest patch index (LPI), contagion (CONTAG) and aggregation index (AI). Additionally, PD and ED were observed to have major impacts on water quality contaminants, particularly in mountainous high flow region (Zone 4), which indicated the fragmentation of forest may not function effectively to decrease overland runoff and consequently let pollutants flow into the river; the landscape metrics of CONTAG was observed to be related to non-point pollutants in mountainous normal flow region (Zone 1) which suggested the correlations between CONTAG and soil erosion and sediment yield; LSI was found an effective indicator for anions in Zone 1, Zone 4 and Zone 5 mainly in high flow season; SHDI metric indicated more water quality variables in each eco-region than SHEI, except in Zone 3 and Zone 4 which only SHEI was observed to contribute to water quality variables. Indicating the variation of landscape heterogeneity contributed to the degradation of water quality in all eco-regions. The results of our study suggested that natural environmental factors such as topography, climate and hydrology may have some impacts on how land use patterns correlate with water quality. Thus, analyzing the relationships between land use patterns and water quality on an eco-functional regionalization basis is considerable. Therefore, further studies are needed to explore how the characteristics of eco-regions affect the casual relationship between land use patterns and water quality.

## Figures and Tables

**Table 1:** Average values of the main environmental variables which characterize the five eco-regions.

Code	Definition	Location	Altitude (m)	Annual Mean Temperature (°C)	Runoff Depth (mm)
Zone 1	Mountainous Normal flow	Great Khingan Mountain	559	−0.26	179
Zone 2	Plain low-flow	Songnen Plain	182	0.36	45
Zone 3	Hilly high-flow	Second Songhua and Songhua river mainstream	332	0.23	303.77
Zone 4	Mountainous high-flow	Changbai Mountain	604	0.26	341.59
Zone 5	Plain normal flow	Sanjiang Plain	174	0.32	177

**Table 2:** Land use and landscape metrics ^a^ used in this study.

Landscape Metrics (Abbreviation)	Descriptions
Arable land (AR)	Land use for crops cultivation, land mainly used for planting and beach cultivated more than three years (unit: %)
Forest (FO)	Including growing arbor, shrub, bamboo, mangrove and other young afforested land.
Grassland (GR)	Land use for herbaceous plant, coverage above 5% (unit: %)
Water (WA)	Inland water area and land use for water conservancy facilities (unit: %)
Urban (UR)	Residential area, industrial area and roads (unit: %)
Unused land (UN)	Barren land, alkaline land, sand and waste land (unit: %)
Patch density (PD)	Numbers of patches per unit area (number per 100 ha)
Largest patch index (LPI)	Percentage of the landscape in the largest patch (unit: %)
Edge density (ED)	Total length of all edge segments per hectare for the considered landscape (unit: m/ha)
Landscape shape index (LSI)	Provides a standardized measure of total edge or edge density that adjusts for size of the landscape.
Contagion (CONTAG)	Tendency of land use types to be aggregated (unit: %)
Interspersion and juxtaposition index (IJI)	Based on patch adjacencies, not cell adjacencies like the contagion index.
Shannon’s diversity index (SHDI)	Based on information theory; indicates the patch density in a landscape (unitless)
Shannon’s evenness index (SHEI)	Minus the sum across all patch types, of the proportional abundance of each patch type multiplied by that proportion, divided by the logarithm of the number of patch types (unitless)
Aggregation index (AI)	Number of like adjacencies involving the corresponding land use type, divided by the maximum possible number of like adjacencies involving the corresponding land use type (unit: %)

^a^ landscape metrics are calculated by FRAGSTATS 4.0.

**Table 3:** Descriptive statistics of landscape metrics in each eco-region in the Songhua River Basin.

Landscape Metrics	Zone 1	Zone 2	Zone 3	Zone 4	Zone 5	One-Way Anova
	Mean	Mean	Mean	Mean	Mean	*p*
PD (#/100 ha)	0.11	0.12	0.10	0.093	0.086	0.002 **
LPI (%)	29.90	37.38	43.82	50.39	25.30	0.007 **
ED (m/ha)	8.19	6.85	6.97	6.86	7.21	0.013 *
LSI	15.57	14.06	14.51	16.55	11.83	0.403
CONTAG (%)	50.79	51.74	55.33	56.59	47.26	0.037 *
IJI (%)	52.38	58.88	53.11	54.04	57.56	0.015 *
SHDI	1.44	1.49	1.38	1.32	1.64	0.040 *
SHEI	0.57	0.58	0.51	0.52	0.63	0.024 *
AI (%)	52.02	60.97	63.89	54.27	62.53	0.136

Abbreviation of landscape metrics are listed in Table 2. * means *p* < 0.05, ** means *p* < 0.01.

**Table 4:** Results from Partial Least Square regression analysis for water quality parameters in each eco-region.

Season		Zone 1			Zone 2			Zone 3			Zone 4			Zone 5		
	Y	R^2^	Q^2^	Component	R^2^	Q^2^	Component	R^2^	Q^2^	Component	R^2^	Q^2^	Component	R^2^	Q^2^	Component
High-flow	pH	0.53	0.51	2	0.58	0.56	2	0.54	0.52	2	0.58	0.56	2	0.54	0.52	2
	EC	0.53	0.51	2	0.73	0.58	3	0.72	0.62	2	0.73	0.58	3	0.72	0.62	2
	DO	0.88	0.60	3	0.72	0.69	3	0.73	0.60	2	0.72	0.69	3	0.73	0.60	2
	COD	0.66	0.57	2	0.73	0.62	2	0.76	0.51	2	0.73	0.62	2	0.76	0.51	2
	COD_MN_	0.63	0.68	2	0.63	0.51	2	0.64	0.58	2	0.63	0.51	2	0.64	0.58	2
	NH_3_N	0.68	0.51	2	0.65	0.62	2	0.70	0.69	2	0.65	0.62	2	0.70	0.69	2
	NO_3_N	0.71	0.65	2	0.63	0.57	2	0.75	0.59	2	0.63	0.57	2	0.75	0.59	2
	TN	0.66	0.51	2	0.72	0.70	2	0.71	0.50	2	0.72	0.70	2	0.71	0.50	2
	TP	0.73	0.66	3	0.71	0.71	2	0.77	0.51	2	0.71	0.71	2	0.77	0.51	2
	F^−^	0.68	0.42	3	0.69	0.58	2	0.81	0.66	3	0.69	0.58	2	0.81	0.66	3
	Cl^−^	0.68	0.55	3	0.49	0.11	1	0.54	0.32	1	0.49	0.11	1	0.54	0.32	1
	${\mathrm{SO}}_{4}^{2-}$	0.52	0.24	2	0.75	0.73	3	0.64	0.52	2	0.75	0.73	3	0.64	0.52	2
Normal flow	pH	0.70	0.52	2	0.59	0.51	2	0.64	0.60	3	0.59	0.51	2	0.64	0.60	3
	EC	0.60	0.50	2	0.82	0.52	2	0.84	0.63	2	0.82	0.52	2	0.84	0.63	2
	DO	0.76	0.59	2	0.79	0.57	2	0.64	0.59	2	0.79	0.57	2	0.64	0.59	2
	COD	0.78	0.59	2	0.61	0.59	2	0.61	0.55	2	0.61	0.59	2	0.61	0.55	2
	COD_MN_	0.63	0.53	2	0.63	0.52	2	0.68	0.63	2	0.63	0.52	2	0.68	0.63	2
	NH_3_N	0.64	0.58	2	0.79	0.60	2	0.60	0.13	2	0.79	0.60	2	0.60	0.13	2
	NO_3_N	0.77	0.69	3	0.72	0.21	2	0.93	0.59	2	0.72	0.21	2	0.93	0.59	2
	TN	0.75	0.68	2	0.80	0.60	2	0.76	0.60	2	0.80	0.60	2	0.76	0.60	2
	TP	0.78	0.80	2	0.73	0.78	2	0.89	0.53	3	0.73	0.78	2	0.89	0.53	3
	F^−^	0.79	0.64	2	0.73	0.69	2	0.64	0.48	3	0.73	0.69	2	0.64	0.48	3
	Cl^−^	0.87	0.74	3	0.47	0.24	1	0.84	0.59	3	0.47	0.24	1	0.84	0.59	3
	${\mathrm{SO}}_{4}^{2-}$	0.68	0.54	2	0.73	0.65	2	0.61	0.60	3	0.73	0.65	2	0.61	0.60	3

**Table 5:** The relative importance of the key variables in the optimal models in high-flow season.

Y	Significant Predictors (*A*)				
	Zone 1	Zone 2	Zone 3	Zone 4	Zone 5
pH	**WA (0.645)**, UR (0.400), SHEI (−0.299)	**FO (−0.440)**, PD (0.079), IJI (0.183)	AR (−0.195), **FO (0.207)**	**GR (0.758)**, UN (−0.365)	UN (−0.071), **LPI (0.938)**
EC	GR (−0.138), **UN (0.222)**, SHDI (0.122)	**UR (0.362)**	**AR (0.180)**, FO (0.152), IJI (−0.179)	**WA (−0.571)**, CONTAG (0.366), AI (−0.220)	AR (0.100), **SHEI (−0.115)**
DO	FO (0.054), **UR (−0.045)**, LPI (0.208)	**GR (0.280)**, UR (−0.210), IJI (0.315)	**AR (−0.235)**, FO (0.190), IJI (0.188)	**FO (0.067)**, GR (0.179), PD (−0.058)	**AR (−0.235)**, UR (−0.387), LPI (−0.073)
COD	LPI (−0.207), **ED (0.264)**, CONTAG (−0.171)	PD (0.080), **IJI (0.081)**, AI (−0.071)	**AR (0.047)**, UN (0.181), PD (0.053)	**WA (−0.494)**, SHEI (−0.201), AI (−0.248)	AR (−0.097), UR (0.126), **SHDI (0.116)**
COD_Mn_	AR (0.209), **ED (0.322)**, CONTAG (−0.172)	AR (0.163), **GR (−0.401)**, UN (0.539)	**FO (0.266)**, WA (−0.371), SHEI (−0.138)	**FO (0.249)**, PD (0.347), ED (0.376)	FO (−0.024), **UR (0.293)**
NH_3_N	GR (−0.367), **SHEI (−0.291)**, AI (−0.289)	LPI (−0.164), **IJI (0.220)**, SHDI (0.079)	**AR (0.255)**, FO (−0.371), LPI (−0.428)	FO (−0.291), PD (0.269), **ED (0.376)**	**UN (0.249)**
NO_3_N	**SHDI (0.259)**, AI (−0.231)	**GR (−0.243)**, LPI (0.254), ED (0.341)	UR (0.311), **LPI (−0.335)**	**UR (0.224)**, PD (0.245), ED (0.175)	**AR (0.289)**, GR (−0.135), SHDI (0.287)
TN	AR (0.253), GR (−0.750), **SHDI (0.396)**	**AR (0.547)**, PD (0.266), IJI (−0.460)	**AR (0.204)**, FO (−0.209)	FO (−0.299), **ED (0.638)**	GR (−0.209), UR (0.451), **SHDI (0.793)**
TP	AR (0.207), **ED (0.736)**, CONTAG (−0.335)	GR (−0.245), **UN (0.553)**, SHDI (−0.357)	**AR (0.278)**, FO (−0.275), IJI (−0.137)	FO (−0.388), PD (0.349), **ED (0.468)**	**AR (0.267)**, LPI (−0.561), SHDI (0.391)
F^−^	SHEI (−0.790), **AI (0.970)**	**GR (−0.206)**, LPI (−0.146)	**AR (0.242)**, FO (−0.228), UR (0.115)	**AR (0.225)**, GR (−0.168)	WA (0.327), LPI (0.472), **LSI (0.410)**
Cl^−^	ED (0.307), IJI (0.588), **SHDI (−0.697)**	**UR (0.478)**, IJI (0.588)	**AR (0.266)**, FO (−0.190), UR (0.444)	UR (0.444), **UN (0.585)**, LSI (0.202)	——
${\mathrm{SO}}_{4}^{2-}$	**AR (0.340)**, WA (−0.462), LSI (−0.206)	——	——	**GR (−0.322)**, WA (0.168)	GR (−0.116), **LSI (0.202)**

Y means the response variables in the PLSR models; A means the regression coefficient; the key variables with the highest VIP values in the optimal models are in bold; “——”means no valid model was found for this water quality variable; Abbreviation of land use/landscape variables are listed in Table 2.

**Table 6:** The relative importance of the key variables in the optimal models in normal flow season.

Y	Significant Predictors (*A*)				
	Zone 1	Zone 2	Zone 3	Zone 4	Zone 5
pH	**WA (0.655)**, UR (0.206), LSI (−0.299)	**GR (0.269)**, SHDI (0.174), AI (0.575)	AR (−0.189), **FO (0.202)**	**SHEI (0.234)**, AI (−0.236)	**UR (−1.167)**, UN (−1.715)
EC	GR (−0.138), **UN (0.302)**, SHDI (0.171)	WA (−0.190), **UR (0.254)**, PD (0.163)	AR (249), **FO (−0.269**), IJI (−0.189)	WA (−0.240), **UR (0.220)**, PD (0.241)	**AR (0.228)**, LSI (0.188), IJI (0.225)
DO	AR (−0.216), **FO (0.211)**, UR (−0.332)	**GR (0.192)**, UR (−0.198), IJI (0.168)	**AR (−0.223)**, FO (0.201), IJI (0.117)	**FO (0.064)**, GR (0.161), PD (−0.124)	**AR (−0.396)**, UR (−0.472), LPI (0.235)
COD	UR (0.306), ED (0.322), **CONTAG (−0.289)**	**UR (0.300)**, AI (0.195)	**UN (0.372)**, SHEI (0.038)	WA (−0.280), **SHEI (−0.225)**, AI (0.257)	**AR (−0.217)**, UR (0.100), SHDI (−0.123)
COD_Mn_	**ED (0.373)**, CONTAG (−0.290)	**GR (−0.503)**, UN (0.694)	LPI (0.168), SHEI (0.017), **AI (0.119)**	**FO (0.181**), PD (0.292), ED (0.291)	**FO (−0.187)**, UR (0.168)
NH_3_N	**UR (0.624)**, SHEI (−0.214), AI (−0.153)	LPI (0.115), **IJI (−0.190)**, SHDI (−0.098)	**GR (0.587)**, LPI (−0.024)	FO (−0.233), PD (0.287), **ED (0.331)**	**UR (−0.202)**, UN (0.225), LSI (−0.195)
NO_3_N	**FO (−0.334)**, SHDI (0.235), AI (−0.254)	**UR (0.169)**, LPI (0.115), ED (0.201)	**UR (−0.291)**, PD (0.188)	UR (0.175), **PD (0.235)**, ED (0.229)	**AR (0.599)**, GR (−0.347), SHDI (0.370)
TN	FO (−0.339), **LPI (0.224)**, CONTAG (0.224)	AR (0.230), PD (0.201), **IJI (−0.367)**	**AR (0.316)**, GR (0.582)	FO (−0.192), **PD (0.349)**, ED (0.435)	**AR (0.705)**, GR (−0.329), SHDI (0.397)
TP	AR (0.317), **ED (0.269)**, CONTAG (−0.256)	UR (0.144), **LPI (0.202)**, SHDI (−0.087)	**AR (0.278)**, FO (−0.213), IJI (−0.121)	FO (−0.426), PD (0.391), **ED (0.448)**	**AR (0.495)**, LPI (−0.329), SHDI (0.347)
F^−^	PD (0.082), **SHEI (−0.272)**, AI (−0.253)	UR (0.225), UN (0.249), **LSI (0.197)**	**AR (0.310)**, FO (−0.274), UR (−0.259)	UR (0.040), **GR (−0.359)**	AR (0.361), LPI (0.059), **LSI (0.091)**
Cl^−^	UN (0.409), **IJI (0.641)**, SHDI (0.962)	**UR (0.429)**, SHDI (−0.388), AI (−0.407)	**FO (−0.300)**,	——	**UR (0.395)**, LSI (0.049), AI (0.101)
${\mathrm{SO}}_{4}^{2-}$	——	**LSI (−0.278)**, SHDI (−0.351), AI (−0.259)	WA (0.247), **PD (−0.408)**	GR (−0.389), WA (0.320), **UR (0.189)**	UR (−0.041), **UN (−0.639)**, LSI (0.319)

Y means the response variables in the PLSR models; A means the regression coefficient; the key variables with the highest VIP values in the optimal models are in bold; “——”means no valid model was found for this water quality variable; Abbreviation of land use/landscape variables are listed in Table 2.

## Appendix A

**Table A1:** The details of all the sampling sites with WGS 84 coordinates.

Number of Sampling Sites	WGS 84 Coordinates of Sampling Sites		Long Term Monitoring Sites
	E	N	
S1	123.78	50.55	Y
S2	123.44	47.96	Y
S3	122.80	47.75	Y
S4	123.46	48.09	Y
S5	124.71	49.49	Y
S6	125.18	49.18	N
S7	125.10	49.12	Y
S8	124.75	48.91	Y
S9	125.51	49.66	N
S10	125.45	49.41	N
S11	122.26	45.92	Y
S12	123.43	46.78	Y
S13	123.6833	46.78333	N
S14	123.86	46.296	Y
S15	123.9167	47.36667	N
S16	126.1054	48.52878	N
S17	125.9574	48.56711	Y
S18	124.53	48.48	Y
S19	124.55	48.23333	N
S20	124.5654	48.36458	Y
S21	125.89	48.0044	N
S22	126.193	48.04149	Y
S23	124.4421	47.5309	Y
S24	124.7294	45.72	N
S25	124.6469	45.43639	Y
S26	124.83	45.16222	Y
S27	124.9833	45.48333	N
S28	125.7	45.335	Y
S29	126.5388	45.75979	N
S30	126.718	45.93	Y
S31	126.72	45.82	Y
S32	126.4161	46.14218	Y
S33	125.357	43.938	Y
S34	125.35	43.85	Y
S35	125.445	44.60313	N
S36	125.6886	44.78805	N
S37	126.1887	44.67113	N
S38	126.0658	44.9	Y
S39	126.48	44.4	Y
S40	126.49	44.31	N
S41	126.9139	46.00074	N
S42	127.0134	45.40921	N
S43	127.0644	44.90426	N
S44	127.3369	46.89167	N
S45	128.1474	45.91833	N
S46	128.6019	47.03444	N
S47	128.8589	47.8175	N
S48	127.873	47.04286	Y
S49	129.3558	48.63694	N
S50	129.4394	46.27222	N
S51	129.5733	46.00361	N
S52	129.58	46.33	N
S53	129.5836	44.53089	Y
S54	129.6389	46.73056	N
S55	129.6722	44.76579	N
S56	130.1581	47.95028	N
S57	128.7394	45.81808	Y
S58	125.3035	44.85627	Y
S59	125.6758	44.76854	Y
S60	125.9005	43.80154	Y
S61	125.7791	44.08246	Y
S62	125.6749	43.56837	Y
S63	126.033	43.30054	Y
S64	127.2599	43.63825	Y
S65	126.43	44.05	Y
S66	126.61	43.78	Y
S67	129.0161	44.06063	N
S68	128.7372	43.75558	N
S69	127.8495	47.11137	N
S70	126.8446	43.11292	N
S71	126.86	43.12	Y
S72	126.1254	42.69658	Y
S73	127.22	42.73	Y
S74	126.98	43.12	Y
S75	128.09	42.36	N
S76	127.7632	42.04037	N
S77	130.5433	46.01944	Y
S78	130.5839	47.28806	N
S79	129.9136	46.64694	N
S80	130.6878	47.03167	N
S81	131.7489	47.23389	N
S82	132.51	47.7	N
S83	132.4581	47.66139	N
S84	132.46	47.724	Y
S85	131.0789	47.67972	N
S86	130.933	45.8141	Y

“Y” means the sample site belongs to long-term monitoring program, while “N” means the sample site is newly setted in this study.

**Table A2:** Correlation matrix of the land use and landscape metrics and population density used in the PLSR analysis ^a^.

Metrics ^b^	AR	FO	GR	WA	UR	UN	PD	LPI	ED	LSI	CONTAG	IJI	SHDI	SHEI	AI
AR	1														
FO	−0.903	1													
GR	−0.231	−0.048	1												
WA	0.219	−0.486	0.045	1											
UR	0.621	−0.527	−0.365	0.096	1										
UN	0.015	−0.254	0.290	−0.025	−0.220	1									
PD	0.310	−0.434	0.239	0.158	0.332	0.202	1								
LPI	−0.252	0.395	−0.216	−0.280	−0.161	−0.215	−0.468	1							
ED	0.196	−0.278	0.284	−0.005	0.206	0.181	0.742	−0.710	1						
LSI	−0.078	0.126	−0.029	−0.383	0.099	0.201	0.048	−0.276	0.434	1					
CONTAG	−0.133	0.368	−0.231	−0.488	−0.08	−0.282	−0.630	0.828	−0.732	−0.112	1				
IJI	0.011	−0.248	0.098	0.547	−0.015	0.283	0.386	−0.502	0.172	−0.167	−0.708	1			
SHDI	0.227	−0.379	0.181	0.245	0.194	0.295	0.502	−0.872	0.722	0.404	−0.831	0.545	1		
SHEI	0.084	−0.298	0.184	0.470	0.042	0.232	0.505	−0.663	0.522	−0.101	−0.856	0.696	0.669	1	
AI	0.022	0.012	−0.161	0.045	−0.021	−0.042	−0.406	0.166	−0.393	0.121	0.276	−0.086	−0.131	−0.620	1

^a^ The bold-faced numerical values indicate a significant relationship at a level of *p* < 0.01. ^b^ Abbreviations for land use and landscape metrics are listed in Table 2.

**Table A3:** Values of regression coefficients from PLSR models describing the relationships between land use/landscape metrics and individual water quality parameters in high flow seasons in each eco-region.

	pH	EC	DO	COD	COD_Mn_	NH_3_N	NO_3_N	TN	TP	F^−^	Cl^−^	$\;{\mathrm{SO}\;}_{4}^{2-}$
*Zone 1 (N = 10)*												
AR		0.173		0.165	**0.209**			**0.253**	**0.207**	0.107	−0.006	**0.340**
FO	0.006	−0.146	**0.054**	−0.199				−0.541			0.044	
GR		**−0.138**				**−0.367**	−0.217	**−0.750**				−0.393
WA	**0.645**		0.064									**−0.462**
UR	**0.400**		**−0.045**			0.098					0.378	−0.323
UN		**0.222**	0.029		0.278		0.205	0.190			0.301	
PD	0.036					0.059				0.336		−0.094
LPI	0.087	0.076	**0.208**	**−0.207**	−0.151				−0.045			
ED			−0.173	**0.264**	**0.246**	0.186			**0.736**		**0.307**	
LSI								−0.605	0.338			**−0.206**
CONTAG			0.170	**−0.171**	**−0.172**	−0.201			**−0.335**		−0.523	
IJI		0.184			−0.076	0.052				0.708	**0.588**	
SHDI		**0.122**	−0.097	−0.052			**0.259**	**0.396**	−0.477		**−0.697**	
SHEI	**−0.299**				0.009	**−0.291**	−0.184			**−0.790**		−0.139
AI	0.261					**−0.289**	**−0.231**	1.101		**0.970**		−0.153
*Zone 2 (N = 23)*												
AR					**0.163**	0.067		**0.547**			0.007	——
FO	**−0.440**			0.047		0.164					−0.034	——
GR	0.231		**0.280**	−0.065	**−0.401**	−0.103	−0.243		**−0.245**	**−0.206**		——
WA		−0.106		−0.029								——
UR		**0.362**	**−0.210**				0.176				**0.478**	——
UN					**0.539**		0.125		**0.553**		0.301	——
PD	**0.079**	−0.017		**0.080**	−0.191	−0.033		**0.266**	0.249			——
LPI				0.053		**−0.164**	**0.254**		0.228	**−0.146**		——
ED		−0.105		−0.054	−0.068	**−0.001**	**0.341**		0.247		0.307	——
LSI										**−0.054**		——
CONTAG				0.059				0.033				——
IJI	0.183		0.315	0.081		**0.220**		−0.460			0.588	——
SHDI	0.023			−0.060	−0.113	**0.079**		−0.368	−0.357			——
SHEI			0.052	−0.044			**−0.342**					——
AI				−0.071								——
*Zone 3 (N = 32)*												
AG	−0.195	0.180	−0.235	0.047		**0.255**		0.204	0.278	**0.242**	0.266	——
FO	0.207	−0.152	0.190	−0.065	0.266	**−0.371**		−0.209	−0.275	**−0.228**	−0.190	——
GR				−0.029								——
WA					−0.371						−0.153	——
UR							**0.311**	0.061		**0.115**	0.444	——
UN				0.181							0.085	——
PD				0.053								——
LPI	−0.105		−0.073		0.169	**−0.428**	**−0.335**		0.100			——
ED	0.062				−0.121				0.012		0.382	——
LSI	0.168		0.180						−0.111		0.202	——
CONTAG		0.106	−0.076			0.123	0.003		0.097			——
IJI		**−0.179**	**0.188**			0.224			**−0.137**	−0.104		——
SHDI	0.022	−0.099	0.063	−0.044	0.041				−0.099			——
SHEI					−0.138	−0.239		−0.107	−0.127			——
AI	−0.048										−0.376	
*Zone 4 (N = 12)*												
AG			−0.037							**0.225**	0.266	0.233
FO			**0.067**		**0.249**	**−** **0.291**		**−** **0.299**	**−** **0.388**	−0.140	−0.189	
GR	**0.758**		**0.179**							**−** **0.168**		**−** **0.322**
WA		**−** **0.571**		**−** **0.494**							−0.153	**0.168**
UR		0.216					**0.224**				**0.444**	
UN	**−** **0.365**	0.187		0.004	0.391			0.268			**0.585**	
PD			**−** **0.058**		**0.347**	**0.269**	**0.245**		**0.349**	0.367		
LPI			0.086							−0.054		
ED			0.057		**0.376**	**0.319**	**0.175**	**0.638**	**0.468**		0.382	
LSI	0.098	0.222	−0.234	0.322							**0.202**	
CONTAG		**−** **0.366**	0.014		−0.146	−0.132			−0.184			
IJI							−0.202					
SHDI	0.316	0.249								−0.171		
SHEI		−0.121	0.371	**−** **0.201**		−0.246	−0.215	−0.167				
AI		**−** **0.220**	0.298	**−** **0.248**			0.081	−0.012			**−** **0.376**	
*Zone 5 (N = 9)*												
AR		**0.100**	**−** **0.235**	**−** **0.097**	0.097		**0.289**		**0.267**		——	
FO					**−** **0.024**	−0.023					——	
GR							**−** **0.135**	**−** **0.209**	−0.160		——	**−** **0.116**
WA			0.092		0.131		0.056			**0.327**	——	
UR			**−** **0.387**	**0.126**	**0.293**			**0.451**	0.235		——	0.105
UN	**−** **0.071**					**0.249**					——	
PD		−0.100	−0.103								——	
LPI	**0.938**		**−** **0.073**		−0.137		−0.182	−0.314	**−** **0.561**	**0.472**	——	
ED		−0.068			0.119						——	
LSI								0.152	0.113	**0.410**	——	**0.202**
CONTAG		0.104	0.023			−0.012					——	
IJI	0.154										——	0.133
SHDI	0.235			**0.287**	0.116		**0.287**	**0.793**	**0.391**		——	
SHEI		**−** **0.115**			0.022	−0.034					——	
AI							−0.090		−0.133	−0.016	——	

The key predictors with the VIP values above 1 in the optimal models are in bold; “——” means no valid model was found for this water quality variable; Abbreviation of land use/landscape variables are listed in Table 2.

**Table A4:** Values of regression coefficients from PLSR models describing the relationships between land use/landscape metrics and individual water quality parameters in normal flow seasons in each eco-region.

	pH	EC	DO	COD	COD_Mn_	NH_3_N	NO_3_N	TN	TP	F^−^	Cl^−^	$\;{\mathrm{SO}\;}_{4}^{2-}$
*Zone 1 (N = 10)*												
AR	0.085	0.167	**−0.216**					−0.297	**0.317**	0.040		——
FO	−0.043	−0.159	**0.211**	−0.128		−0.050	**−0.334**	**0.339**	−0.023	−0.075		——
GR		**−0.138**	0.259								0.025	——
WA	**0.655**						0.282	0.221	−0.237	0.138	−0.392	——
UR	**0.206**	0.081	**−0.332**	**0.306**		**0.624**	−0.006		−0.073	0.066		——
UN		**0.302**	−0.182								0.409	——
PD			−0.046	−0.036	0.066	0.288	−0.009	0.077		**0.082**	1.024	——
LPI		0.160	0.184	−0.217			0.251	**0.431**	−0.100			——
ED		−0.089	−0.169	**0.322**	**0.373**		−0.229	−0.215	**0.269**	−0.220		——
LSI	**−0.299**	−0.079	0.076			−0.299	0.132		0.094	0.019		——
CONTAG				**−0.289**	**−0.290**		0.166	**0.224**	**−0.256**	0.216	0.841	——
IJI		0.235		−0.013	0.066	0.245	0.170	0.103		−0.078	**0.641**	——
SHDI	0.243	**0.171**		−0.055			**0.235**		0.041	0.097	**0.962**	——
SHEI			0.098	−0.184	−0.120	**−0.214**		−0.076		**−0.272**	−0.904	——
AI	0.321		0.032	−0.140	−0.086	**−0.153**	**−0.254**	−0.026		**−0.253**		——
*Zone 2 (N = 23)*												
AR								**0.230**		−0.023	0.240	
FO										0.076	−0.151	0.189
GR	**0.269**		**0.192**	−0.136	**−0.503**	−0.073	0.165		−0.058	−0.062		
WA	0.249	**−0.190**	0.169			−0.119					−0.203	−0.039
UR		**0.254**	**−0.198**	**0.300**	0.111	−0.111	**0.169**		−0.098	**0.225**	**0.429**	
UN	−0.397		−0.127		**0.694**	0.083	0.344		**0.144**	**0.249**	−0.238	
PD	−0.028	**0.163**	−0.057	−0.113		−0.008	0.192	**0.201**	0.043	−0.265		
LPI	−0.186		−0.099	0.020		**0.115**	**0.115**		**0.202**	−0.176		
ED	0.003		0.013	−0.013	−0.055	0.025	**0.201**	0.212	0.003		0.069	
LSI			0.214							**0.197**		**−0.28**
CONTAG	−0.083	0.046	−0.058	0.035		0.056		0.058	0.094			
IJI		−0.048	**0.168**	−0.161		**−0.190**		**−0.367**	−0.157			
SHDI	**0.174**		0.085	−0.069		**−0.098**	−0.053		**−0.087**		**−0.388**	**−0.35**
SHEI	−0.289	0.056	0.046			−0.024	−0.219		−0.073			0.210
AI	**0.575**			**0.195**	0.050		0.249		0.007		**−0.407**	**−0.259**
*Zone 3 (N = 32)*												
AR	**−0.189**	**0.249**	**−0.223**		0.136		−0.049	**0.316**	**0.278**	**0.310**		
FO	**0.202**	**−0.269**	**0.201**		−0.147		0.095	−0.178	**−0.213**	**−0.274**	**−0.300**	
GR						**0.587**		**0.582**		0.191	0.134	
WA				−0.190						−0.013		**0.247**
UR	−0.123						**−0.291**			**−0.259**		
UN		0.152		**0.372**						0.112		
PD		−0.181			−0.193		**0.188**			−0.181		**−0.408**
LPI	−0.104	0.009	−0.114	0.153	**0.168**	**−0.024**	−0.180	−0.037		−0.020	0.120	−0.107
ED	0.054	−0.065								0.014		
LSI	0.069											
CONTAG	−0.082	0.135	−0.065	0.007	0.024	0.320		0.169	0.100		0.112	
IJI	0.093	**−0.189**	**0.117**	−0.234				−0.328	**−0.121**			
SHDI	0.078	0.049	0.087	0.025	−0.049	0.307	0.144			0.042	−0.045	0.206
SHEI	0.086	−0.111	0.081	**0.038**	**0.017**	−0.291		−0.032	−0.129		−0.107	
AI	−0.029	0.018	−0.039		**0.119**					0.015		
*Zone 4 (N = 12)*												
AR		0.062			**0.074**	0.038		0.004	0.062			
FO		−0.036	0.064		**−0.181**	−0.233		0.192	−0.426	0.069		
GR	−0.124	−0.020	0.161	0.188						−0.359		−0.389
WA		−0.240		−0.280			−0.198	−0.102				0.320
UR		0.220		−0.126	**0.125**	0.017	0.175		0.023	0.040		0.189
UN	−0.054			0.205				−0.160				
PD	0.044	0.241	−0.12	0.130	**0.292**	0.287	0.235	0.349	0.391			−0.05
LPI	0.026	−0.092	0.065				−0.057	−0.053	−0.018	−0.065		−0.135
ED	0.028	0.232	−0.098	0.112	**0.291**	0.331	0.229	0.435	0.448			−0.050
LSI		0.142	−0.045	0.194		−0.170			−0.153			
CONTAG	−0.027	−0.125	0.073	−0.109	**−0.256**	−0.227	−0.051	−0.111	−0.227	−0.230		0.009
IJI					**0.316**		−0.203	−0.224				
SHDI	−0.056	0.057				0.057		0.014	−0.007			0.149
SHEI	0.234		−0.239	−0.225			−0.150					
AI	−0.236			0.257								0.051
*Zone 5 (N = 9)*												
AR		**0.228**	**−0.396**	**−0.217**			**0.599**	**0.705**	**0.495**	**0.361**		−0.344
FO		0.033		−0.018	**−0.187**	−0.051			−0.151	0.103	−0.023	0.178
GR		−0.038		0.058		0.037	**−0.347**	**−0.329**	−0.014	−0.082	−0.026	
WA				0.057		0.015			0.078	−0.049	−0.058	
UR	**−1.167**		**−0.472**	**0.100**	**0.168**	**−0.202**		0.135	−0.065	−0.298	**0.395**	**−0.041**
UN	**−1.715**	−0.204				**0.225**	−0.469		−0.467		0.191	**−0.639**
PD	−0.420			0.005	0.176	−0.016			0.038	0.022	−0.083	
LPI		0.130	**0.235**	−0.153		−0.084			**−0.329**	**0.059**	0.147	
ED		−0.039		0.064	0.079	0.030			0.146	−0.016	−0.114	0.118
LSI		**0.188**				**−0.195**			0.199	**0.091**	**0.049**	**0.319**
CONTAG		0.059		−0.002		−0.071			0.103	0.025	0.064	0.121
IJI	−0.434	**0.225**	0.098			−0.156	0.011	0.023	−0.006		−0.026	
SHDI			0.180	**−0.123**			**0.370**	**0.397**	**0.347**	0.086	0.022	
SHEI		−0.056		−0.036		0.080			−0.229	−0.023	−0.035	−0.232
AI		0.081		−0.058		−0.072			−0.063	0.035	**0.101**	−0.011

The key predictors with the VIP values above 1 in the optimal models are in bold; “——” means no valid model was found for this water quality variable; Abbreviation of land use/landscape variables are listed in Table 2.
